# The Durham Initiative for Stomach Health (DISH): a pilot community-based *Helicobacter pylori* education and screening study

**DOI:** 10.1186/s12876-020-01405-w

**Published:** 2020-08-06

**Authors:** Sydnee Crankshaw, Julia Butt, Jennifer M. Gierisch, Nadine J. Barrett, Sabrena Mervin-Blake, Kevin Oeffinger, Steven Patierno, Valarie Worthy, Ronald Godbee, Meira Epplein

**Affiliations:** 1 Cancer Control and Population Sciences Program, Duke Cancer Institute, Durham, NC USA; 2 Department of Population Health Sciences, Duke University School of Medicine, 2424 Erwin Road, Suite 602, Durham, NC 27705 USA; 3 Center of Innovation to Accelerate Discovery and Practice Transformation, Durham Veterans Affairs Medical Center, Durham, NC USA; 4 Department of Family Medicine and Community Health, Duke University School of Medicine, Durham, NC USA; 5 Duke Clinical and Translational Science Institute, Duke University School of Medicine, Durham, NC USA; 6 Department of Medicine, Duke University School of Medicine, Durham, NC USA; 7 Department of Pharmacology and Cancer Biology, Duke University School of Medicine, Durham, NC USA; 8 The River Church, Durham, NC USA

**Keywords:** Cancer prevention, Stomach cancer, Helicobacter pylori, Education, Screening

## Abstract

**Background:**

Approximately 15% of all cancers are due to infection. The bacteria *Helicobacter pylori* is the single leading carcinogenic infectious agent and the main cause of stomach cancer. Prevalence of *H. pylori*, and, correspondingly, stomach cancer incidence and mortality, is significantly greater among African Americans than whites in the United States. In the present study, we conducted a pilot community-engaged *H. pylori* education and screening study in partnership with a predominantly African American church in Durham, North Carolina.

**Methods:**

Initially, we consulted with community advisory boards and convened stakeholder meetings with local community members and primary care physicians. We then developed this pilot study through an iterative collaboration with church partners. Our main outcomes were feasibility and acceptability as measured by participation in a one-day *H. pylori* screening initiative, and participation in follow-up for those who tested positive. We also sought to determine prevalence and determinants of active *H. pylori* infection in this population.

**Results:**

Community engagement informed the event logistics, messaging, educational materials provided, and follow-up plans. A total of 92 individuals participated in the primary study event, 25% of whom had a current *H. pylori* infection. Of those, 87% returned for the follow-up events, among whom 70% had successfully cleared their infection.

**Conclusions:**

Through community engagement, community-based *H. pylori* screening and stomach cancer prevention is feasible and acceptable. This is a necessary step in order to move stomach cancer prevention forward to population-based precision *H. pylori* screening and eradication.

## Background

Infection with the bacteria *Helicobacter pylori* (*H. pylori*) is the primary cause of stomach cancer, the 6th leading cause of death from cancer among African American men. Stomach cancer also accounts for the greatest difference in death rates between African American men and women, compared to non-Hispanic white men and women [1]. Approximately 30% of Americans are chronically infected with *H. pylori*, with rates above 50% for men and women of color, including African Americans, Asian Americans, and Hispanics [2, 3]. Currently, there is no systematic *H. pylori* screening and eradication plan in the United States to prevent stomach cancer or other *H. pylori*-associated diseases (gastritis, peptic ulcers, and MALT lymphoma), even though *H. pylori* eradication therapy has been shown to be highly effective and feasible [4]. Moreover, clinical trials have found that treatment for *H. pylori* (generally 14 days of two antibiotics plus a proton pump inhibitor and/or bismuth therapy) reduces stomach cancer risk by up to 50% [5]. Population-wide screening and treatment for *H. pylori* has also been estimated to be substantially cost-effective, even in lower-risk countries like the United States [6, 7].

There are multiple barriers to conducting population-wide screening and treatment for *H. pylori* that may hinder large-scale eradication of this bacteria and diminish the promise of meaningful reductions in the rates of stomach cancers. Concerns arise as only a small percentage of individuals with *H. pylori* will go on to develop stomach cancer, and eradication treatment requires the use of multiple antibiotics which are not consistently effective due to increasing *H. pylori* antibiotic resistance and lack of medication adherence. As we ultimately seek to establish population-based precision prevention of stomach cancer through *H. pylori* eradication, we recognize that the first step is to engage the communities at highest risk to educate and support *H. pylori* screening for those who seek to reduce their stomach cancer risk.

We developed the Durham Initiative for Stomach Health (DISH), a community-engaged pilot study to assess the feasibility of an *H. pylori* screening and eradication approach. The ultimate aim is to reduce stomach cancer incidence in populations in Durham County, North Carolina, at highest risk, including African Americans. Durham is a majority minority city with African Americans comprising 39.7% of the population, Asians 5.2%, Whites 48% and other races 7.5% [8]. For the present project, we sought to engage the local community to build and implement a stomach cancer education platform that included screening for *H. pylori* and motivating *H. pylori*-positive participants to seek treatment through conversation with their primary care providers.

## Methods

### Community and stakeholder engagement

DISH was originally designed as a multi-phase study with initial work intended to provide *H. pylori* testing to those at highest risk, primarily African Americans, in the local Durham community. A summary of the initial planning, final project processes, and lessons learned, is shown in Table 1, and described in detail below.

**Table 1:** DISH Community and Stakeholder Engagement and Impact on Study Procedures

	Initial project ideas	Final project processes	Impact of Study
**Stakeholder meetings**			
Input from health services & community- based researchers	One-on-one meetings with select faculty	Roundtable meeting with experts from throughout the continuum of community outreach to policy implementation	Group feedback enhanced a bigger-picture thinking of the overall goals, towards which the current project would be a first step
Input from community	Development of a steering committee	Work with established community advisory councils	Capitalizing on already existing relationships with the community is pragmatic and feasible
Input from clinicians	Focus groups with clinicians	One-on-one meetings with clinicians	Clinicians have little time, so arranging one-on-one meetings on their schedules is more practical
**Study planning**			
How to approach potential study participants	Meet with church pastor	Meet with church pastor; present during Sunday services; meet with congregants	Importance of meeting the congregation and introducing the topic personally, and presenting the project as a cancer prevention strategy rather than focus on disparity
How to describe the study	Study flyer	Study flyer, study brochure, in-person meetings at the church to explain the project	Interacting with potential study participants in multiple ways allows for an iterative process to best share study information
**Participant recruitment**			
Location	Clinics	Community (at a local church)	Individuals are most comfortable at sites they frequent and trust
No. of events and sites	Multiple dates and sites	One-day event on site at one church	Scaling down to for ease of execution and assessment of logistics
Date and timing of event	Sunday after services	All-day Tuesday prior to evening services	Provides flexibility for potential participants
Consent	In-person consent	Electronic + in-person consent	To make the event logistics work more smoothly, make as many tasks available to be completed prior to the day of event as possible
Study enrollment	In-person enrollment	Online + in-person enrollment	(see above)
**Study event**			
Questionnaire	Potentially relevant gastric cancer risk factors, plus detailed lifestyle variables	Shorten as much as possible - remove religiosity questions, but add questions to help think about long-term implementation	Participants need to feel that the questions are reasonable, not invasive (like religiosity questions), but that also get to the larger issues of beliefs/behavior, physician interaction, and finances
Biospecimen Collection	Breath test, blood draw, stool sample	Breath test and blood draw	The stool sample would not have added significantly more information, but would create an additional barrier to participation.
Participant reimbursement	Amazon gift card	Walmart gift card plus boxed meal, social security number waiver received	Walmart was favored by this community; participation during lunch or dinner hour highlighted importance of boxed meal; requiring a social security number provides an additional barrier.
**Follow-up**			
Individual results	No return of individual results	Results mailed to participant with an accompanying phone call by study team within 2 weeks of study event	There is value and need to give back to participants. Follow-through includes: staff phone-calls to results, patient navigators provided to those with financial barriers, and physician executive summary to inform guideline-concordant *H. pylori* treatment.
Re-testing	No re-testing	Follow-up events at church to re-test after treatment	There is documented ~ 30% failure of *H. pylori* treatment to eradicate; re-testing allows us to re-visit the community, confirm eradication or to support seeking of salvage treatment.

As part of initial planning, we intended to gather input from three groups of stakeholders: health services and community-based researchers; community members; and local clinicians. Early meetings with the Recruitment Innovation Center, a center focused on optimizing recruitment into clinical studies, and the Community Engaged Research Initiative, a community-engaged capacity building core, both part of the Duke Clinical Translational Science Institute (CTSI), provided critical input suggesting that the DISH study team should seek out community collaborations with groups who have existing relationships with Duke clinical research. The Duke Cancer Institute’s Office of Health Equity (OHE) had an existing 4-year relationship with a local, predominantly African American church. This church is led by a pastor strongly interested in improving the health of his community, and thus a potential good partner for DISH. This local church had previously collaborated with Duke Cancer Institute (DCI) to raise awareness around strategies to prevent and treat cancer, including educational events on prostate and colon cancer, clinical research and trials seminars, and health fairs.

In parallel with these conversations, DISH staff engaged with other key community members. The study principal investigator (ME) and research program leader (SC) presented the DISH protocol to both the African Methodist Episcopalian (AME) Zion Health Equity Advocates & Liaisons (AME Zion HEAL) partnership and the DCI Office of Health Equity’s Community Advisory Council to introduce the study, explain eligibility, enrollment, study procedures, and populations of interest, and most importantly, to gain insight from these community leaders on study details. The AME Zion HEAL is a partnership between 17 predominantly African American AME Zion churches and the Duke Clinical Translational Science Institute (CTSI). The goal of AME Zion HEAL is to increase health equity and improve health in the African American community through a partnership designed to cultivate trust and increase engagement in clinical research among the African American community. The DCI OHE Community Advisory Council is a vital component of the health disparities work of the DCI and is comprised of representatives from public and private agencies, community members, and persons concerned with the cancer needs and disparities in Durham and across North Carolina.

From these meetings valuable information was gained regarding study processes and questionnaire details, which allowed us to amend the data collection tools accordingly, as shown in Table 1. One-on-one meetings were also held with primary care physicians to gain knowledge about *H. pylori* treatment and policy, and moreover to attempt to understand their varied clinical perspectives regarding our proposed project, which includes activation of *H. pylori*-positive individuals with their primary care providers. From these interactions with clinicians, we determined the need for a one-page Physician Executive Summary, including approved treatment regimens, to accompany the results of a positive *H. pylori* test that the study participants could then share with their provider.

### Study planning

Reflecting DISH team goals, and informed by key stakeholders, it was determined that the first phase of DISH would be a screening and education project onsite at a church that was acquainted with clinical research, beginning with a one-day enrollment event. OHE facilitated a meeting of DISH staff with the church leader as described above that allowed the DISH principal investigator and research program leader to visit the church to meet with the pastor and begin the conversation about *H. pylori*, stomach cancer, and health disparities, as well as the feasibility of hosting a study event at his church. From this initial meeting, common ground on the mutual goals of improving the health of the congregation was established, aiding in the building of trust and partnership. The pastor also gave invaluable insight into the presentation and communication of the study to the congregation, emphasizing that the DISH team should focus on the aspect of the study that allows individuals to make a positive step in reducing their stomach cancer risk, rather than on the statistics of racial disparity for this disease. The pastor and study team also agreed on the necessity of a commitment to the partnership, including follow-up after the initial study event was complete. Thus, with the full partnership of the pastor and critical input into the study approach, this church was chosen to be the flagship site for the study.

One week prior to the established date for the enrollment event, DISH staff attended Sunday church services to meet the congregation, introduce the topic of *H. pylori* and its role in gastric cancer development, explain the study, inform interested congregants how to sign up for the event, and hand out flyers. The DISH flyer included information about the study, including the web address to the online enrollment link (see Supplemental Fig. 1). This allowed potential participants to access the link to determine eligibility, view and sign the e-consent, and fill out the study questionnaire. As informed by stakeholders, the website was created to reduce the amount of time each participant spent at the enrollment event. The church pastor also posted information regarding the enrollment event on his social media pages and posted a video discussing the event with the principal investigator on his YouTube channel*.*

### Participant recruitment

Decisions regarding participant recruitment were made in collaboration with the pastor of the church. Specifically, the one-day event to screen for *H. pylori* would happen on-site at the church, where potential participants would be most comfortable, and would take place on a Tuesday, from 1 pm to 9 pm prior to, during, and after church services that evening. While we had originally planned for the event on a Sunday, church partners advised that potential participants were unlikely to stay after services (which end at lunchtime), and a longer, weekday event, would allow for greater flexibility for participants. Concern about the logistics of many people showing up at one time, but needing first to go through the informed consent process, as well as fill out a lengthy questionnaire, led to the development of an e-consent as well as online questionnaire that individuals could complete prior to the one-day study event. We also decided to provide tablets at the church for individuals to utilize on-site, and had paper copies of all materials (consent and questionnaire) available for those who were more comfortable completing the documents in that fashion.

Inclusion criteria for participation included age 40 or older and with the ability to provide informed consent in English. Exclusion criteria related to the ability for a breath test for assessment of *H. pylori* infection to produce accurate results, and included the use of antibiotics, Pepto Bismol, or any proton pump inhibitor in the past 2 weeks prior to enrollment, as well as prior gastric surgery and/or gastric cancer. At the study event, after meeting inclusion/exclusion criteria and consenting, individuals were screened for *H. pylori* infection with a breath test, as well as asked to complete a questionnaire and donate a blood sample. The blood sample was analyzed using multiplex serology for secondary analyses [9]. The results of the *H. pylori* breath test were then mailed to participants within 2 weeks of the enrollment event, and DISH staff followed up with all *H. pylori*-positive individuals by phone within 3 months, and with an additional breath test 6 months later. A second follow-up event was added 2 months later to ensure that all *H. pylori*-positive participants who were interested and could not attend the 6-month event were re-tested.

### Study event

#### Study procedures

The DISH study event was planned for Tuesday, May 15, 2018 in one large room at the church. On the day of the event, study staff met with community members when they entered the study room to confirm the participants met the eligibility criteria, had reviewed and signed the study consent, and had completed the study questionnaire. Individuals who completed some or all of these documents online, as well as individuals who had not visited the study website previously, were welcome to participate. As necessary, DISH staff directly administered the questionnaire with study participants.

After consent was obtained and the study questionnaire begun, participants moved to various, marked tables for 1) the blood draw and 2) the breath test. Study staff administered the breath test and nurses/phlebotomists drew the blood sample. In between participants completed the questionnaire if necessary, and/or waited at tables with fellow participants. After participants completed all study procedures, they received a gift card and boxed dinner, as participants had to fast for an hour prior to taking the breath test, as required by the manufacturer.

#### Breath test

The *Helicobacter pylori* Urea Breath Test, Infra-red (UBiT®), is a non-invasive, non-radioactive method for detecting urease associated with *H. pylori* infection by measuring labelled carbon dioxide in the subject’s breath. To take the breath test, the participant exhaled into a balloon-like bag for a baseline sample, then drank a small amount of a lemon-flavored solution, which included 13C-urea – urea labeled with a non-radioactive carbon isotope. The participant remained seated for 15 min, after which they exhaled into another balloon-like bag. After sample collection, the bags with the exhaled breath samples were mailed to Quest Diagnostics for testing. An increase in the ratio of 13CO2 to 12CO2 between pre- and post-ingestion samples indicates the presence of *H. pylori*-associated urease [10].

#### Questionnaire

The 12-page questionnaire sought to capture all potentially relevant gastric cancer risk factors, plus detailed lifestyle variables. A list of the variables included in the baseline questionnaire is provided in Table 2.

**Table 2:** Questionnaires

Category	Variables
***Baseline Questionnaire***	
Demographics	Age, race, ethnicity, education, income, occupation
Gastric Symptoms	Stomach ache, heartburn, acid reflux, hunger pains, nausea, rumbling in stomach, feeling bloated, burping, passing gas, constipation, diarrhea, loose stools, hard stools, urgent need to have bowel movement, not completely emptying bowels
Family History	Cancer, 1st degree relatives, if occurred before age 50; stomach ulcers, gastritis, *H. pylori*
Medication Use	Use of antibiotics, aspirin, acetaminophen, peptic ulcer medication, pills for diabetes, insulin, allergy pills, asthma
Medical History	Heart trouble, high blood pressure, anemia, asthma, hayfever, skin allergy, food allergy, emphysema, COPD, stomach ulcer, duodenal ulcer, IBD, gastritis, *H*. *pylori*, GERD, heartburn, pancreatitis, TB, hepatitis, HPV, MS, diabetes
Cancer Screening	Women (pap smear, mammogram, sigmoidoscopy, colonoscopy), men (digital rectal exam, PSA blood test, sigmoidoscopy, colonoscopy)
Smoking	Smoking status, number per day, age first smoked, number of years; chewing tobacco, snuff/dip, pipes, cigars
Alcohol	Current use, type of drink, amount and how often
Physical Activity	Average sleep during week/week-end, vigorous and moderate activity, walking, sitting
Height and weight	Self-reported height/weight
***Follow-up Questionnaire***	
Gastric Symptoms	*Same as those in the baseline questionnaire (*Table 2*above)*
Family History	Any 1st degree relatives during the past six months being diagnosed with cancer, ulcers, gastritis, *H. pylori*
Medical History	Changes in the past 6 months regarding anemia, stomach ulcer, duodenal ulcer, IBD, gastritis, GERD, diabetes
Interaction with doctor	Hp + only - communication with doctor about infection (in person, over the phone, via email), timeline from diagnosis to doctor’s visit, did doctor re-test prior to treatment, did doctor retest after treatment, what was prescribed (number of pills/day/duration), adherence to prescribed medication, cost
Experience with physician	Scale of very poor, poor, fair, good, very good; friendliness, explanation about their condition, concern about questions/worries, involvement in decision making, information about medications, follow-up care, amount of time spent with provider, confidence in provider, recommend provider
Height and weight	Self-reported height/weight

To measure current gastric symptoms, the Gastrointestinal Symptom Rating Scale (GSRS) was used. The GSRS is a disease-specific instrument used to evaluate common symptoms of gastrointestinal disorders. This scale contains 15 questions with a seven-point Likert scale ranging from no discomfort to very severe discomfort. These fifteen questions were then broken down into four categories – abdominal pain (stomach pain, hunger pains, nausea), reflux syndrome (heartburn, acid reflux), diarrhea syndrome (diarrhea, loose stools, bowel urgency), indigestion syndrome (rumbling, bloating, burping, passing gas) and constipation syndrome (constipation, hard stools, feeling of incomplete evacuation) [11].

Study data were collected and managed using REDCap electronic data capture tools hosted at Duke University [12].

#### Follow-up

Within 2 weeks after the study event, participants were mailed a letter informing them whether they had a current *H. pylori* infection. If the report was a positive breath test, the letter explained what is known about the association of infection with *H. pylori* and gastric cancer risk, along with a recommendation that the individual speaks with their primary care physician. *H. pylori*-positive individuals also received a “Physician Executive Summary,” to share with their primary care provider, which listed approved *H. pylori* eradication therapy treatments. Producing a counseling document to the physician that outlines treatment options can produce higher eradication rates, and was recommended during one of the one-on-one preparatory meetings DISH staff had with a primary care physician. Moreover, there is evidence that counseling of primary care providers improves appropriate *H. pylori* treatment and eradication rates [13, 14]. Participants also received a toll-free number in case they had questions that needed immediate attention. Additionally, participants were informed that if they did not have a regular primary care physician or health insurance, DISH staff would put them in touch with a patient navigator from OHE.

Approximately 3 months after the initial enrollment event, *H. pylori* positive participants were contacted by phone to ask if they had sought treatment. Additionally, participants identified at this point who were without insurance were referred to patient navigators from OHE to link them with physicians who could treat their infection at no or a reduced cost.

After the initial enrollment, two separate follow-up events were held at the church (one at 6 months, the other at 8 months). These follow-up events were for participants who tested positive for *H. pylori*, along with a sample of *H. pylori*-negative participants, to determine the percent treated, and among those, the percent successfully eradicated by a follow-up breath test, as well as to collect information on *H. pylori*-positive participants’ interactions with their primary care providers. Information on financial costs incurred by the participants was also requested. Those individuals found to be *H. pylori*-positive at the follow-up event were given a second letter and physician summary to bring to their primary care physician. A more detailed listing of variables in the follow-up questionnaire is given in Table 2.

## Results

### Participant accrual

**Fig. 1:**
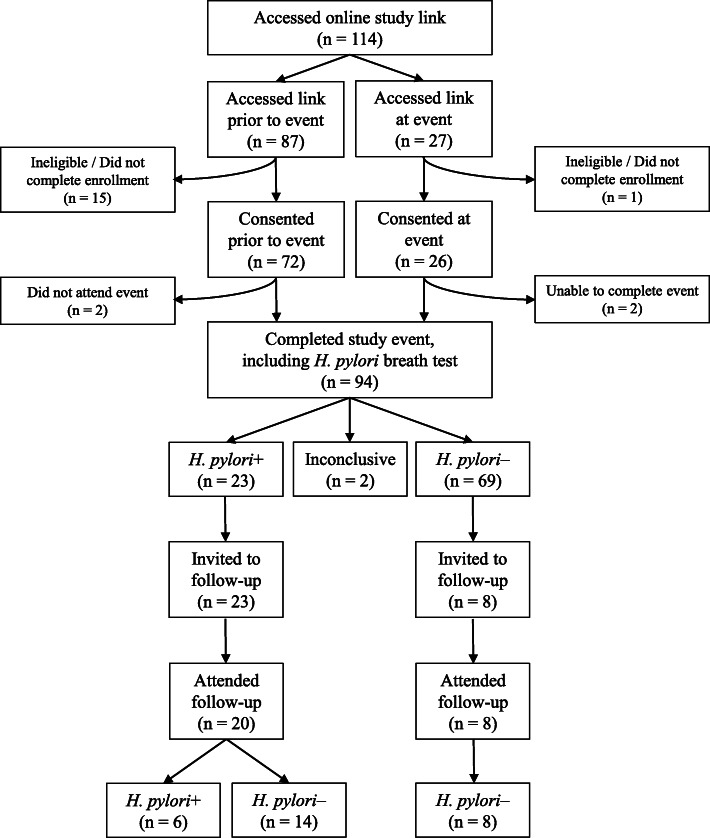
Flowchart of the DISH Study

As illustrated in Fig. 1, 114 community members accessed the online enrollment link in an effort to begin the eligibility and consenting process, as well as to fill out the study questionnaire. Eighty-seven accessed the online enrollment link prior to the DISH event, with 72 of the 87 (83%) completing the eligibility, consent and questionnaire prior to the event.

In total, 94 individuals successfully participated in the enrollment event, close to our goal of 100 participants. Of the 94 who participated, two had inconclusive breath test results and were not included in the analyses of results. These two community partners were offered retesting, and one did come back for a second breath test, but it too was inconclusive. Inconclusive breath test results occur in 1–2% of a patient population [15].

### Participant characteristics

Of the 92 participants included in the analyses of *H. pylori* prevalence, the majority identified as African American (97%), female (79%), never smokers (79%), not current drinkers (64%), having an associate’s degree or higher (53%), an annual income of $50,000 or more (54%), and with health insurance (90%). Most participants had never been regular aspirin users (67%), and while participants reported high blood pressure (54%), anemia (37%), and allergies (30%), the prevalence of other chronic health conditions was relatively low in this population (see Table 3).

**Table 3:** Study participant characteristics

	All *N* = 92)	*H. pylori* negative *N* = 69)	*H. pylori* positive *N* = 23)
Age			
Mean (SD)	53.9 (8.9)	53.4 (8.9)	55.6 (8.6)
Range	(40.8–76.6)	(40.8–71.9)	(43.1–76.6)
Sex, N (%)			
Female	73 (79.3)	57 (82.6)	16 (69.6)
Male	19 (20.7)	12 (17.4)	7 (30.4)
Race, N (%)			
African American	89 (96.7)	66 (95.7)	23 (100.0)
Other	3 (3.3)	3 (4.3)	0 (0.0)
Smoking, N (%)			
Ever	19 (20.7)	16 (23.2)	3 (13.0)
Never	73 (79.3)	53 (76.8)	20 (87.0)
Alcohol use, N (%)			
Currently	33 (35.9)	27 (39.1)	6 (26.1)
Never	31 (33.7)	23 (33.3)	8 (34.8)
Used to	28 (30.4)	19 (27.5)	9 (39.1)
Education, N (%)			
No high school degree	2 (2.2)	2 (2.9)	0 (0.0)
High school but no college degree	41 (44.6)	27 (39.1)	14 (60.9)
Associates degree or higher	49 (53.3)	40 (58.0)	9 (39.1))
Income, N (%)			
Missing	3	1	2
< $10,000	4 (4.5)	4 (5.9)	0 (0.0)
≥ $10,000–$24,999	4 (4.5)	4 (5.9)	0 (0.0)
≥ $25,000–$49,999	41 (46.1)	30 (44.1)	11 (52.4)
≥ $50,000–$74,999	15 (16.9)	10 (14.7)	5 (23.8)
≥ $75,000–$100,000	15 (16.9)	13 (19.1)	2 (9.5)
> $100,000	10 (11.2)	7 (10.3)	3 (14.3)
Health insurance, N (%)			
No	9 (9.8)	7 (10.1)	2 (8.7)
Yes	83 (90.2)	62 (89.9)	21 (91.3)
BMI, N (%)			
≤ 25	10 (10.9)	9 (13.0)	1 (4.3)
> 25–30	26 (28.3)	17 (24.6)	9 (39.1)
> 30–35	27 (29.3)	19 (27.5)	8 (34.8)
> 35	29 (31.5)	24 (34.8)	5 (21.7)
Regular aspirin use, N (%)			
Current	12 (13.0)	6 (8.7)	6 (26.1)
Former	18 (19.6)	14 (20.3)	4 (17.4)
Never	62 (67.4)	49 (71.0)	13 (56.5)
Medical history, N (%)			
High blood pressure	50 (54.3)	36 (52.2)	14 (60.9)
Anemia	34 (37.0)	29 (42.0)	5 (21.7)
Allergies	28 (30.4)	22 (31.9)	6 (26.1)
Asthma	14 (15.2)	11 (15.9)	3 (13.0)
Type 2 diabetes	16 (17.4)	13 (18.8)	3 (13.0)
GERD	11 (12.0)	9 (13.0)	2 (8.7)
Heartburn	8 (8.7)	6 (8.7)	2 (8.7)
IBD	5 (5.4)	5 (7.2)	0 (0.0)
Gastritis	5 (5.4)	4 (5.8)	1 (4.3)
Stomach ulcer	5 (5.4)	5 (7.2)	0 (0.0)

### *H. pylori* prevalence

Overall, 25% of participants with a conclusive breath test were determined to have a current *H. pylori* infection (see Fig. 1). As shown in Table 3, there were no significant differences between *H. pylori*-positive and -negative individuals by any demographic or medical history characteristics.

### 3-month phone follow-up

Three months after the main DISH event, study staff sought to re-contact all 23 *H. pylori*-positive participants to learn of the participants’ follow-on actions, specifically communicating with their primary care providers and seeking treatment. DISH staff ultimately made contact with 74% of the *H. pylori*-positive participants (17 of 23), and 82% (14 of 17) reported having seen their doctor and being prescribed medication for their infection. DISH staff used this opportunity to encourage those *H. pylori*-positive individuals who had not yet sought treatment to speak with their primary care physician. Two of the three participants who had not sought care were referred to patient navigators through OHE.

### In-person follow-up events

All 23 *H. pylori*-positive participants and a subset of *H. pylori*-negative participants (*n* = 8), for comparison, were invited back for the DISH follow-up events to re-test for active *H. pylori* infection. Twenty (87%) of the *H. pylori*-positive participants participated in the follow-up events along with all 8 (100%) of the *H. pylori*-negatives. The three *H. pylori*-positive participants who did not attend were contacted and offered a second breath test, with one returning for the follow-up breath test at a later date. At the follow-up events, 70% (14 of 20) of *H. pylori*-positive participants had their infection eradicated, leaving 30% (6 of 20) with a persistent *H. pylori* infection (see Fig. 1). All *H. pylori*-negative participants at the initial event who were re-tested remained negative at the follow-up events.

### Follow-up survey

As part of the follow-up events, participants were asked if there were any changes within the past 6 months regarding their personal medical history or family history of cancer, ulcers, or gastritis. One *H. pylori*-negative participant stated a family member had recently been diagnosed with ulcers. All others answered these questions as ‘none diagnosed.’

Physician interaction questions were based on a 5-point Likert scale from the Press Ganey Patient Satisfaction Survey with values of very poor, poor, fair, good, and very good [16]. In general, *H. pylori*-positive individuals reported positive experiences during their visit to request treatment for their infection, with the majority reporting good or very good experiences to the physician interaction questions (see Table 4). However, only a small percentage of *H. pylori*-positive individuals (3 of 20) reported that their provider followed up with them after treatment to confirm eradication, which is necessary to provide guideline-concordant care according to the American College of Gastroenterology [4].

**Table 4:** Experience with care provider among *H. pylori*-positive individuals who sought care

Variable	% reporting good or very good experience
Confidence in provider	100%
Likelihood of recommending this provider to others	95%
Explanations the provider gave about *H. pylori* infection	95%
Information the provider gave about medications	95%
Concern the provider showed for patient’s questions or worries	90%
Provider used words they could understand	90%
Provider included them in decision about their treatment	85%
Friendliness/courtesy of the provider	75%
Amount of time provider spent with them	74%
Instructions about follow-up care	72%

Participants were again asked the gastric distress questions at follow-up. There were no significant differences by *H. pylori* status or changes from the initial event to the six-month follow-up event for any gastric distress symptom, among those with cleared, persistent, or no infection.

## Discussion

With 92 participants and 87% follow-up of *H. pylori*-positive individuals, this pilot *H. pylori* screening and eradication study, conducted with an engaged community partner, demonstrated feasibility and acceptability.

The success of this study was supported through active collaboration with multiple community stakeholders. Specifically, the knowledge and understanding gained from all who participated in planning the study – community groups, clinicians, health community-based researchers – prior to its initiation enabled a larger understanding of the situation in which the project would be conducted, and ultimately informed all aspects of the study materials, logistics, and follow-up. We had many iterations to our study documents based on input from these meetings, making the data collection tools more acceptable and relevant to our community and intended user. Importantly, partnering with a community group that had previously been introduced to the DCI and its research, including a pastor who is very involved in the health promotion of his congregants and the larger community, enabled a setting of trust that allowed for an on-site clinical study.

We found that building trust and creating relationships with the community was essential when implementing a study that involves biospecimen collection and the sharing of personal information. It is also necessary to set aside adequate time to talk about the study with potential participants and keep those lines of communication open during and after the study. Most importantly, we found that engaging with the participants to fully understand their needs, concerns, and experiences is critical. For example, one participant has had stomach distress for years, resulting in a hospital stay where she was asked to take the breath test for *H. pylori*, but at the time refused because the process and rationale of the breath test – drinking a yellow-colored liquid and blowing into a bag – was not clearly communicated to her. After our church engagement, testing, and recommendations, she received treatment, eradicated her *H. pylori,* and now feels significantly better. Moreover, we plan to return to the church with the overall results as presented here, to communicate the important role our community partners played in this successful pilot project.

Overall, 23% of participants in DISH were found to have an active *H. pylori* infection, which is below the national average estimates of 30% for the general population and 50% for African Americans [3]. While the prevalence of *H. pylori* in this community was lower than expected from national statistics, this could be attributed to the study population differing from a more general African American population. For example, this study population including a larger representation of women who are often seen to be less likely to be infected with *H. pylori* [2, 17], and suggesting that greater effort is needed to encourage men to participate in screening. This population was also composed of individuals of higher average socio-economic status, who are less likely to be infected with *H. pylori*.

As only 70% of *H. pylori*-positive DISH participants successfully eradicated their bacteria, additional research is needed to address the issues of antibiotic resistance and medication adherence, as well as physician follow-up [18, 19]. While our eradication rate was below the estimated national norm of 80% [20], it has been suggested that eradication could be up to 95% effective if there is patient adherence and treatment based on knowledge regarding local *H. pylori* antibiotic resistance [21]. During our study, one participant determined to have a persistent *H. pylori* infection met with their physician who decided to perform an endoscopy, and subsequently found multiple ulcers. The physician sent the tissue to be tested for antibiotic resistance and found that the participant’s *H. pylori* was resistant to clarithromycin. Because of this, a different therapy was prescribed. The most recent endoscopy showed the ulcers healing and the patient has cleared their *H. pylori* infection. The challenge of medication adherence is illustrated by one DISH participant, who, unaware of the adverse effects, stopped taking the prescribed medication 4 days early and was found to have a persistent infection during the DISH follow-up. Approximately 50% of patients with a chronic illness do not take medications as prescribed, which can contribute to increased health care costs as well as negative health outcomes for patients [22, 23]. In our follow-up surveys, we were unable to detect the reason for lack of eradication among the other participants, suggesting the concerns nationally of *H. pylori* antibiotic-resistant strains and physician and/or patient non-adherence to treatment and/or medication guidelines may be the main causes.

A lack of physician follow-up is an additional challenge. The American Clinical Guidelines recommendation is to test patients after treatment therapy for eradication determination, but it is estimated that only 20 to 40% of primary care physicians confirm *H. pylori* eradication, with only slightly higher rates among gastroenterologists [18, 24, 25]. However, education of clinicians to this clinical guideline can improve retesting for eradication [13].

## Conclusions

In conclusion, the DISH study sought to assess the acceptability and feasibility of an *H. pylori* education and screening study, to more finely characterize the prevalence of infection with *H. pylori* in an African American church-based population in the local Durham, North Carolina area, and the opportunities for targeted eradication therapy. We have demonstrated that by engaging with stakeholders in an iterative fashion, and building trust and cultivating relationships with potential study populations, such a study is indeed possible. This pilot study also illustrated remaining issues to be resolved prior to the initiation of larger eradication trials. Specifically, these issues include improving *H. pylori* eradication rates through increasing antibiotic resistance-informed treatment, patient medication adherence, and physician follow-up to confirm eradication, barriers that need to be addressed by clinicians and researchers within and beyond the context of minority health.

## Supplementary information

**Additional file 1: Supplemental Figure 1.** DISH Study Flyer.

## Data Availability

The datasets generated and/or analysed during the current study are not publicly available due to the small numbers and a publicly identified community partner, but are available from the corresponding author on reasonable request.

## Abbreviations

AME Zion HEAL: African Methodist Episcopalian Zion Health Equity Advocates & Liaisons
CTSI: Clinical Translational Science Institute
DCI: Duke Cancer Institute
DISH: Durham Initiative for Stomach Health
GSRS: Gastrointestinal Symptom Rating Scale
*H. pylori*: *Helicobacter pylori*
OHE: Office of Health Equity
