# Supplementary material for: The Durham Initiative for Stomach Health (DISH): a pilot community-based Helicobacter pylori education and screening study
Source: BMC Gastroenterol. 2020 Aug 6;20:261. doi: 10.1186/s12876-020-01405-w (PMC7409393; doi:10.1186/s12876-020-01405-w)
Supplement: Supplementary file 1 — Additional file 1: Supplemental Figure 1. DISH Study Flyer. [file 12876_2020_1405_MOESM1_ESM.pdf]

Partner in Research to Improve Your Stomach Health!

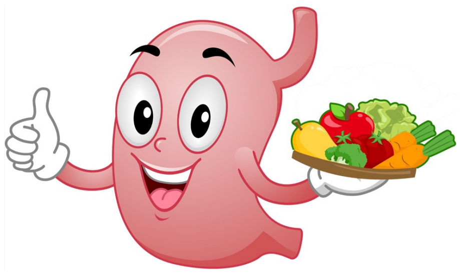

DISH
Durham Initiative
for Stomach Health

What is DISH?

The Durham Initiative for Stomach Health (DISH) is a study to share information on the risks of stomach cancer and a bacteria called *Helicobacter pylori* (*H. pylori*) that commonly lives in the stomach, to ultimately reduce the burden of stomach cancer in the Durham community and **to improve stomach health for all.**

Who can participate?

DISH is open to anyone who:

- Is 40 years old or older
- Has not had prior stomach surgery
- Has not had stomach cancer
- Has not used antibiotics, pepto bismol, or any proton pump inhibitor (like Prilosec) in the past 2 weeks

What will I be asked to do?

- Complete a questionnaire
- Give a small blood sample
- Complete a breath test for *H. pylori* status
- Participate in a follow-up if *H. pylori* positive or among those selected who are *H. pylori* negative

★ Compensation will be provided to eligible participants.

How do I learn more?

- Contact the study leaders at meira.epplein@duke.edu or Sydnee.crankshaw@duke.edu
- Call the study team at 919-681-4762
- Determine eligibility and consent to the study via the following link:

http://cancer.duke.edu/DISH

Upcoming DISH event at The River Church May 15, 2018 from 2-8pm

4900 Prospectus Dr, Durham, NC 27713

DISH Study
2424 Erwin Road, Suite 602
Room 6027

Phone: 919-681-4762
Fax: 919-681-4766
Email: sydnee.crankshaw@duke.edu
